# Quotation accuracy in medical journal articles—a systematic review and meta-analysis

**DOI:** 10.7717/peerj.1364

**Published:** 2015-10-27

**Authors:** Hannah Jergas, Christopher Baethge

**Affiliations:** 1 University of Cologne Medical School, Cologne, Germany; 2 Department of Psychiatry and Psychotherapy, University of Cologne Medical School, Cologne, Germany; 3 Deutsches Ärzteblatt & Deutsches Ärzteblatt International, Cologne, Germany

**Keywords:** Quotation accuracy, References, Bibliography, Meta-analysis, Systematic review, Medical journals, Journalology, Citations, Impact factor

## Abstract

**Background.** Quotations and references are an indispensable element of scientific communication. They should support what authors claim or provide important background information for readers. Studies indicate, however, that quotations not serving their purpose—quotation errors—may be prevalent.

**Methods.** We carried out a systematic review, meta-analysis and meta-regression of quotation errors, taking account of differences between studies in error ascertainment.

**Results.** Out of 559 studies screened we included 28 in the main analysis, and estimated major, minor and total quotation error rates of 11,9%, 95% CI [8.4, 16.6] 11.5% [8.3, 15.7], and 25.4% [19.5, 32.4]. While heterogeneity was substantial, even the lowest estimate of total quotation errors was considerable (6.7%). Indirect references accounted for less than one sixth of all quotation problems. The findings remained robust in a number of sensitivity and subgroup analyses (including risk of bias analysis) and in meta-regression. There was no indication of publication bias.

**Conclusions.** Readers of medical journal articles should be aware of the fact that quotation errors are common. Measures against quotation errors include spot checks by editors and reviewers, correct placement of citations in the text, and declarations by authors that they have checked cited material. Future research should elucidate if and to what degree quotation errors are detrimental to scientific progress.

## Introduction

Citations are an essential and defining element of scientific manuscripts. Ideally, they confirm statements by the authors or refer to work important to the understanding of a text and, thus, enable readers to understand the context of an article. In scientific communication in medicine citations are indispensable for explaining the rationale or the conclusions of a study, or for the argument of a review paper. Insofar as transparency of scientific reasoning is important as a means and an end of evidence based medicine, correct quotations are a vital part of evidence based communication. Also, citations form the basis of most relevant evaluation metrics for academic performance, e.g., Impact factor, Hirsch factor, or SCImago Journal & Country Rank.

Several studies have investigated the extent to which quotations in medical articles support authors’ claims or whether they are inaccurate (an overview is provided by Wager & Middleton (2008)). Results differ: for example, in what is probably the first study on the subject, De Lacey and co-workers (1985) reported that 15% of 300 references contained quotation errors, that is errors of content as opposed to mere formal errors in citation, such as errors in author names or page references (reference or citation errors). The authors considered 6,3% of all references “seriously misleading” for the reader. One of the more recent studies (Luo et al., 2013), however, found major discrepancies between author statement and source cited (“major error”) in 14.5% of all references.

Wager & Middleton (2008) summarized the research published until mid-2007, but we are not aware of a systematic and current review or of a meta-analysis estimating quotation accuracy in medicine. Also, earlier work did not take into account the role of indirect references or different statistical approaches to measuring quotation accuracy.

As a consequence, we carried out a systematic literature review and meta-analysis of studies on quotation accuracy in medical journal articles. Citation accuracy—the correctness of bibliographic information—is not the subject of this study. We aimed at estimating the rate of quotation errors in the field of medicine and at finding out how many of the errors were major—i.e., not at all coherent with a claim by citing authors—and how many were minor.

## Methods

This is a systematic review and meta-analysis investigating quotation accuracy.

### Eligibility criteria

We searched for all studies analyzing quotation accuracy in medical journal articles. Quotation accuracy, for the purposes of this study, is defined as the correctness of the content of a literature reference, that is, whether the reference supports or is in accordance with the statement by citing authors. Accuracy of medical quotations in other contexts, for example, quotations on the web, such as references in Wikipedia (Azer, 2014), or in print advertisements (Del Signore et al., 2011) was not considered in this project.

### Literature search

We searched *Medline* and *PubMed Central* (via PubMed) from their inception through December 26, 2014 using the following search algorithm:

(accura* OR inaccura* OR error OR mistake OR correct* OR incorrect*) AND (citation* OR quotation* OR reference* OR source OR bibliography) AND (bibliography as topic[MeSH] OR periodicals as topic[MeSH])

We did not exclude grey literature. No language restrictions applied. HJ and CB independently screened titles and abstracts, read full-texts if needed for assessment, and discussed unclear cases. Reference lists of studies so-included and of reviews on the topic were hand-searched. In addition, based on a *Web of Science* search, all papers that cited one of the studies included were screened.

### Data collection

Results describing quotation accuracy were documented in an Excel spreadsheet. Meta data (e.g., first author, year of publication, medical specialty) and study results were independently collected by HJ and CB, and differing evaluations were reconciled by discussion. Authors were approached by e-mail if data were incomplete or if full texts had not been published.

We documented the proportion of wrong quotations. Three measurements of quotation errors dominate the literature, all of which were collected as stated in the articles: two of them use the number of references as denominator, i.e., the number of sources referred to, and one is based on quotations, i.e., the number of citations in the text. The numerator is constituted by the number of false quotations, but reference based aproaches differ with respect to the maximum number of errors per reference: in some studies this number is restricted to one error per reference, in another group of studies researchers counted all errors per reference. In this analysis the approaches are named “reference based, restricted,” “reference based, unrestricted,” and “quotation based.”

We divided quotation errors into two grades, more serious and less serious ones, as did almost all authors in the field, often using the term major and minor errors. For example, De Lacey, Record & Wade (1985) defined a severely flawed quotation as “seriously misrepresenting or bearing no resemblance to the original source.” More recently, Luo et al. (2013) graded errors as major “if the reference contradicted, failed to substantiate, or was irrelevant to the author’s assertion in the article.” The guiding principle of these and other definitions is that a major quotation error is not at all in accordance with the claim of the authors. Minor errors, on the other hand, are often defined as inconsistencies and factual errors not severe enough to contradict a statement by citing authors. Again, in De Lacey and co-workers’ (1985) definition, “this group covered quotations that misled or could mislead, but the errors were not sufficiently serious to destroy or fundamentally to alter the meaning of the source.” In collecting data we followed the definition by study authors as long as it was not in conflict with these guiding principles. If a reference error was so severe that it was impossible for the researchers to track down a source, we subtracted such references from the denominator, if the authors had not already done so. We also collected the proportion of total errors.

Some researchers consider indirect references minor quotation errors. Indirect, or secondary, references are defined as citations to other sources than the original, for example, a citation to a review paper instead of the research article. Data was collected as to whether a study had included or excluded secondary references as minor quotation errors.

### Outcome criteria

The main outcome was the proportion of major, minor and total quotation errors. We conducted one meta-analysis across all studies (main analysis). In the event of more than one approach of quotation error management in a given study, the default was the unrestricted reference based approach allowing for more than one error per reference (in Forest plots main analysis data sets are denoted by a 2 behind the first author of the study, e.g., “Davids 2”). In the event studies presented a breakkdown of minor errors including the number of secondary references secondary references were included in the main analysis.

### Sensitivity and subgroup analysis

Analyses were carried out for each approach to determining quotation accuracy separately: reference based, restricted and unrestricted, and quotation based. In another sensitivity analysis minor and total quotation rates were calculated without secondary references if study reports specified those numbers. Additional subgroup analyses were carried out with origin of the study (surgical vs. non surgical specialty) and number of raters (1 vs. >1) as moderator variables. In a meta-regression we searched for an association between publication date of a study on quotation accuracy and major as well as total quotation error rate. Both subgroup analyses and the meta-regression are based on data from the main analysis.

### Risk of bias

The risk of bias of individual studies was assessed using random selection of references (yes vs. no) and number of indepedent raters (1 vs. >1) as variables. The risk of bias was considered low if there was positive evidence from a study report that references were randomly selected for evaluation and that a minimum of two researchers had independently rated the material. Otherwise, the risk of bias was assumed to be high—even if authors did not refer to randomization or the number of independent raters in the manuscripts (We considered it unlikely that such important design elements were simply forgotten in the report).

### Data analysis

Results are presented as percentage of errors with 95% confidence intervals. Due to the heterogeneous nature of the papers included we employed random effects models in all meta-analyses. Heterogeneity is documented by I^2^-values. Subgroup analysis and meta-regression are based on mixed effects models. We used a funnel plot and Egger’s test to detect evidence for publication bias. Further, we removed all studies one by one from the main analysis in order to test the robustness of results. For analysis we used Excel, Open Meta Analyst, and Comprehensive Meta Analysis, Version 2.

### Additional analysis

A small group of studies analyzed the accuracy of quotation of a specific source article (as opposed to the prevailing design of investigating the accuracy of quotations to a set of different references). These studies were not part of the main analyses or of sensitivity and subgroup analyses but are presented seperately. Since these authors selected a particular index article that may have come to their attention precisely because of quotation difficulties, inclusion may have introduced bias. Owing to the small number of studies we did not summarize estimates using meta-analysis.

## Results

**Figure 1:**
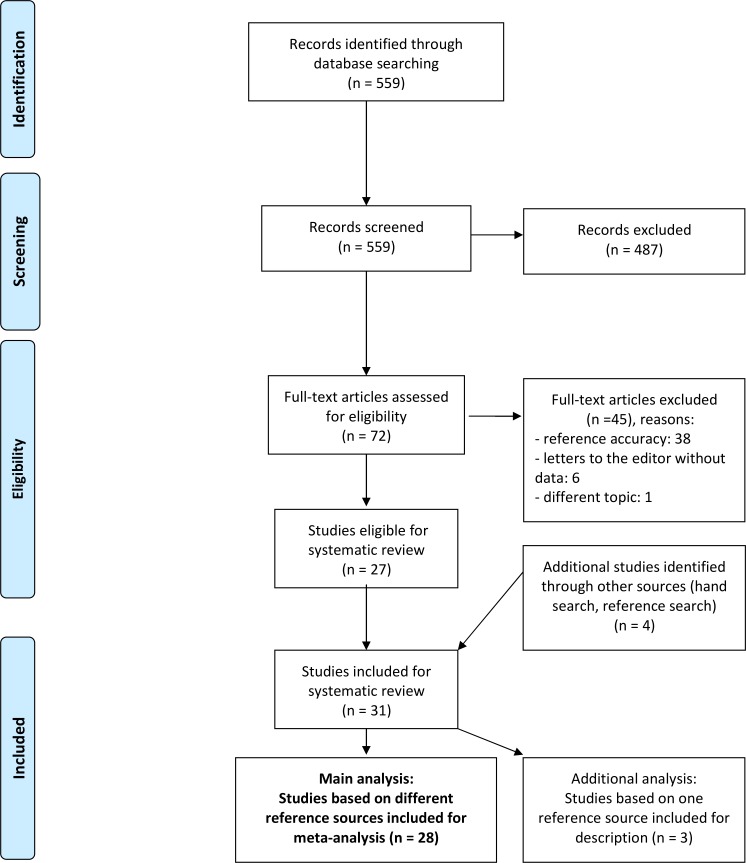
PRISMA flowchart.

We screened 559 papers in our Pubmed search, out of which 72 full texts were read resulting in the inclusion of 27 studies. From other sources we included four additional studies. In total, we included 28 studies in our main analysis (De Lacey, Record & Wade, 1985; Lowry, 1985; Eichorn & Yankauer, 1987; Neihouse & Priske, 1989; Evans, Nadjari & Burchell, 1990; Hobma & Overbeke, 1992; Puttermann, 1992; Goldberg et al., 1993; George & Robbins, 1994; Hansen & McIntire, 1994; Warren et al., 1997; Schulmeister, 1998; Lawson & Fosker, 1999; Lee & Lee, 1999; Fenton et al., 2000; Pieters, Ceysens & De Heyn, 2001; Gosling, Cameron & Gibbons, 2004; Lukić et al., 2004; Buchan, Norris & Kuper, 2005; Gupta et al., 2005; Reddy et al., 2008; Al-Benna et al., 2009; Singh & Chaudhary, 2009; Awrey et al., 2010; Davids et al., 2010; Mertens & Baethge, 2011; Buijze et al., 2012; Luo et al., 2013) and three in an additional analysis (see Fig. 1 for PRISMA flowchart). Except for two articles in Dutch (Hobma & Overbeke, 1992; Pieters, Ceysens & De Heyn, 2001) and one in German (Mertens & Baethge, 2011), all studies were published in English. The studies appeared between 1985 and 2013, covered a wide range of medical specialties (detailed in Table 1) and, in total, reported on the analysis of 7,321 references (range: 46–2011, median: 164.5).

**Table 1:** Study characteristics.

First author	Year of publication	Study size^a^	Risk of bias analysis— evidence of		Risk of bias	Medical specialty
			Randomisation	>1 indep rater		
Al-Benna	2009	113	1	0	0	Surgery
Awrey	2010	900	1	1	1	Surgery
Buchan	2003	200	1	1	1	Ophthalmology
Bujize	2011	2011	1	1	1	Orthopedics
Davids	2010	200	1	1	1	Orthopedics
De Lacey	1985	300	1	1	1	General medicine, mixed specialties
Eichorn	1987	150	1	1	1	Public health
Evans	1990	137	1	1	1	Surgery, gynecology
Fenton	2000	153	1	0	0	Otorhinolaryngology
George	1994	239	1	0	0	Dermatology
Goldberg	1993	145	1	1	1	Emergency medicine
Gosling	2004	320	1	0	0	Manual therapy
Gupta	2005	176	0	1	0	Pediatrics
Hansen	1994	95	1	0	0	Radiology
Hobma	1992	99	1	0	0	General medicine
Lawson	1999	147	1	0	0	Psychiatry
Lee	1999	200	1	0	0	Dermatology
Lowry	1985	61	0	0	0	General medicine
Lukic	2004	199	1	1	1	Anatomy
Luo	2013	249	1	1	1	Orthopedics
Mertens	2011	50	1	1	1	General medicine
Neihouse	1989	99	1	0	0	Pharmacology
Pieters	2001	95	1	1	1	Psychiatry
Puttermann	1992	120	1	0	0	General medicine
Reddy	2008	255	1	1	1	Surgery
Schulmeister	1998	180	1	1	1	Nursing
Singh	2009	46	1	0	0	Dermatology
Warren	1997	382	0	0	0	Infectious diseases

**Notes.**

Risk of bias 0 high 1 low

a Number of references/quotations as used for main analysis (quotation error rates as presented in the paper and using unrestricted reference based quotation error data as default if more than one approach was reported).

In 25 studies, references for analysis were retrieved by some kind of randomization and in 15 studies a minimum of two researchers independently rated quotations for accuracy. As a result, we rated 14 studies as carrying a low risk of bias (De Lacey, Record & Wade, 1985; Eichorn & Yankauer, 1987; Evans, Nadjari & Burchell, 1990; Goldberg et al., 1993; Schulmeister, 1998; Pieters, Ceysens & De Heyn, 2001; Lukić et al., 2004; Buchan, Norris & Kuper, 2005; Reddy et al., 2008; Awrey et al., 2010; Davids et al., 2010; Buijze et al., 2012; Mertens & Baethge, 2011; Luo et al., 2013; see Table 1).

### Main analysis

**Figure 2:**
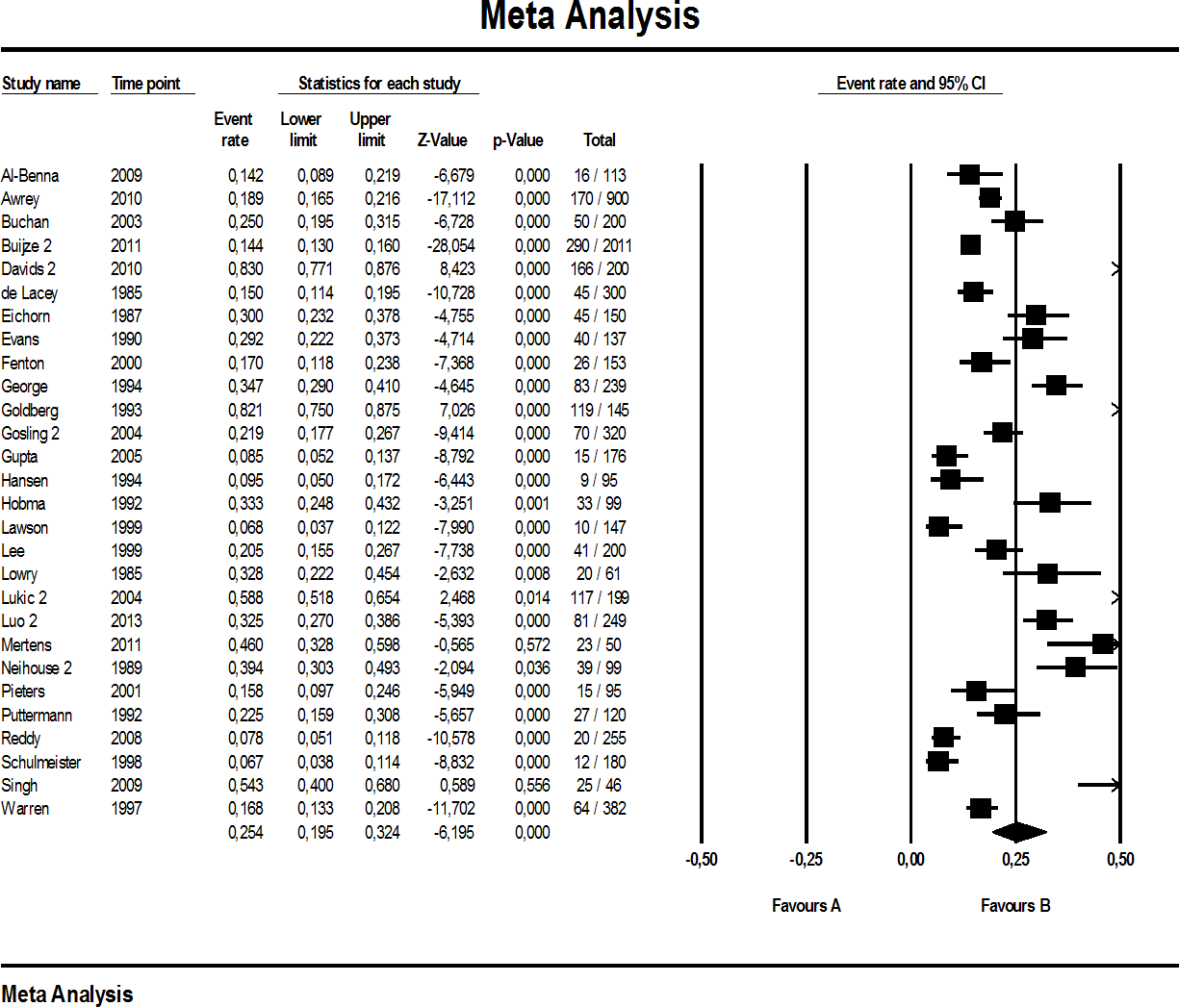
Forest plot of total quotation errors (main analysis). A “2” behind first author names indicates use of data relevant for our main analysis. In supplementary analyses below the studies appear without a number, indicating use of data subsets for sensitivity anlyses (see methods).

**Figure 3:**
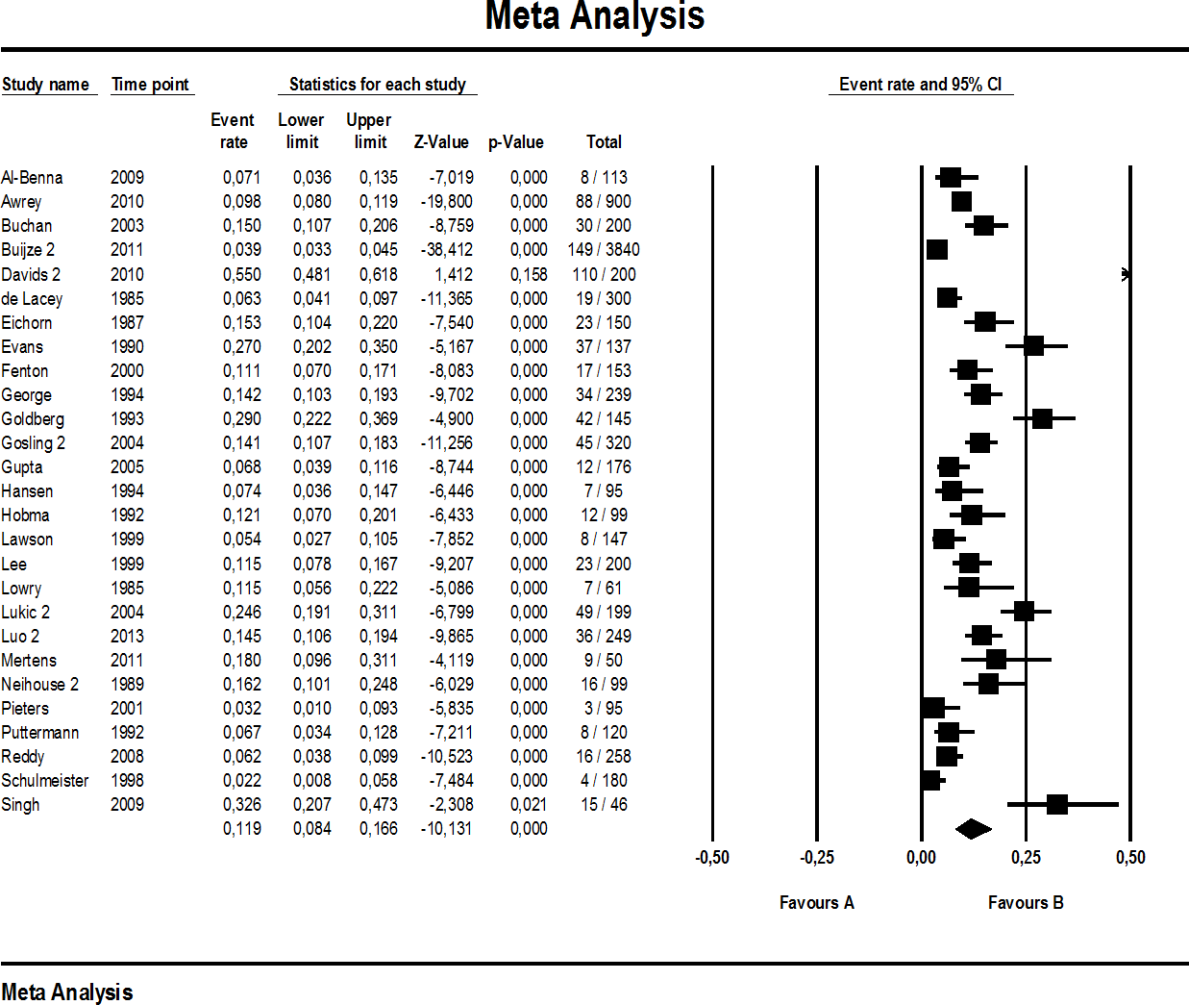
Forest plot of major quotation errors (main analysis).

**Figure 4:**
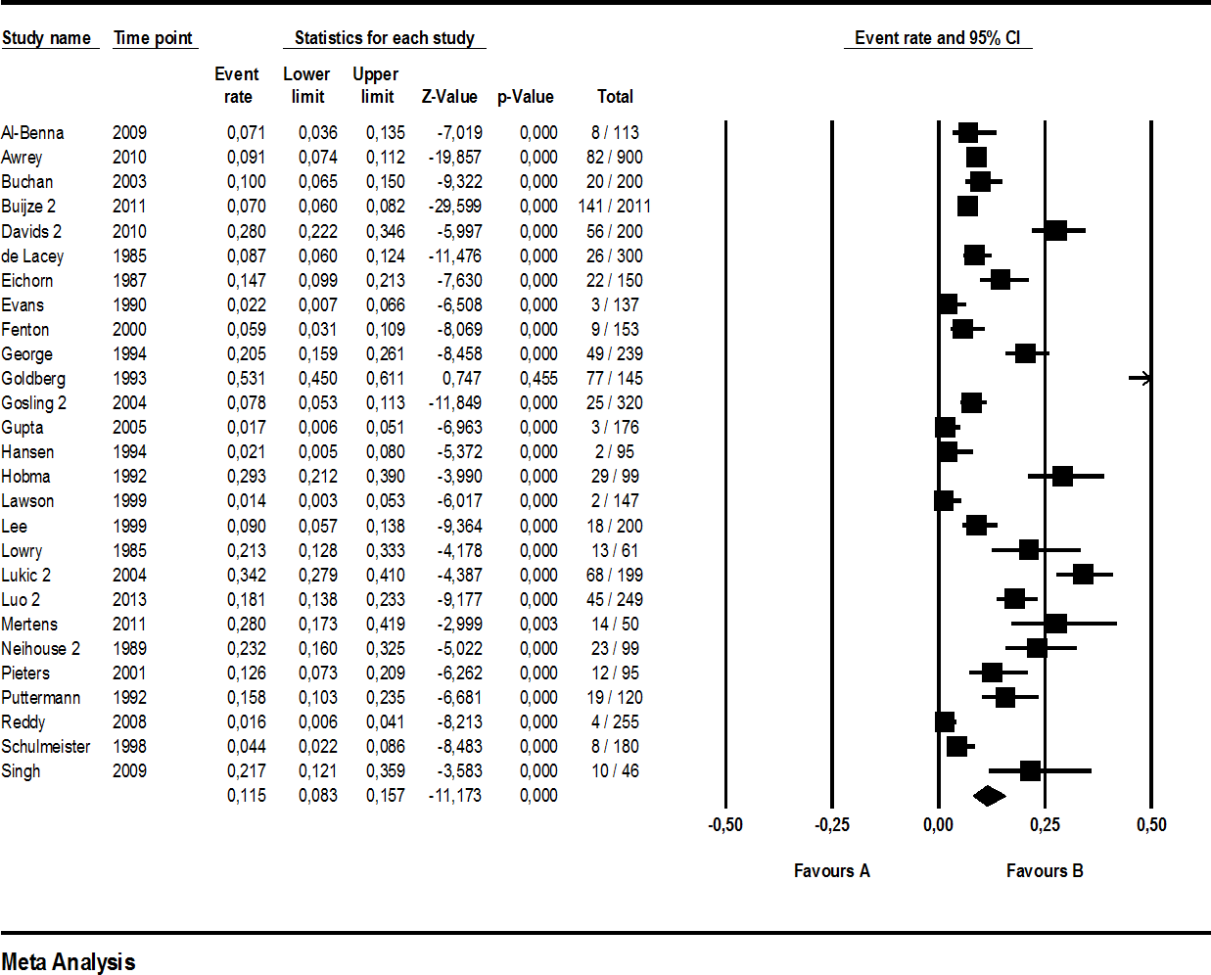
Forest plot of minor quotation errors (main analysis).

The proportion of major errors reported in the investigations selected ranged 2.2%–55.0% (median: 11.5%, *n* = 27), whereas minor errors ranged from 1.4% to 53.1% (median: 9.6%, *n* = 27). Total quotation error rate reached from 6.7% to 83.0% (median: 22.5, *n* = 28). A study-by-study breakdown including all events (quotation errors) and study sizes is provided for the main analysis in Figs. 2–4.

Roughly one in every eight to nine references was seriously incorrect, according to our main analysis (11.9% major quotation errors [8.4, 16.6]), with considerable heterogeneity among the 27 studies included (I^2^: 95%). A similar picture emerged for minor quotation errors, including secondary references: 11.5% [8.3, 15.7], I^2^: 95% (*n* = 27 studies). Regarding total quotation errors, we estimate from the studies included that one fourth of all references is wrong or problematic (25.4% [19.5, 32.5], *n* = 28). Again, heterogeneity was large (I^2^: 97%).

### Sensitivity, subgroup analyses and meta-regression

Seperately analyzing all three statistical aproaches to evaluating quotation accuracy revealed similar results: estimates for major errors ranged from 11.6% to 12.3%, for minor errors 10.0%–12.2%, and for total errors 21.8–26.1. Heterogeneity remained large with I^2^-values between 82% and 98%. Table 2 and Figs. S1–S11 provide complete results.

**Table 2:** Percentages of errors in quotations of scientific articles in medicine. Main analysis and sensitivity analyses. Estimates are based on references (the denominator is the number of references in an article) or on quotations (the denominator is the number of quotations in a text. According to studies from this systematic review (Buijze, Davids, George, Gldberg, Lukic, Luo, Neihouse), the average reference is quoted about 1.7 times in an article (range: 1.37–1.99). Estimates based on references can differ in the number of errors counted per reference: only 1 or >1 (“reference based, restricted” vs. “reference based, unrestricted”). Main analysis is based on all studies. If more than one approach was reported the default was >1 error counted on the basis of references (reference based, unrestricted). Random effects models were employed in all analyses. Indirect references: references to a secondary source, such as a review article instead of the original article. Low vs. high risk of bias analysis is based on main analysis. Total *N*: 7,171 references. 95% confidence intervals in square brackets, number of studies, and I^2^-statistic as measurement of heterogeneity.

	Main analysis	Sensitivity analyses				
		Reference based, restricted	Reference based, unrestricted	Quotation based	Without indirect references	Low vs. high risk of bias
**Major errors,** % [95% CI] Studies, I^2^-statistic	**11.9 [8.4, 16.6] 27 studies, I^2^: 95%**	**12.3** [9.3, 6.1] 15 studies, I^2^: 82%	**11.6** [6.1, 20.8] 12 studies, I^2^: 98%	**11.9** [6.9, 19.9] 6 studies, I^2^: 97%	not applicable^a^	**12.6** [7.1, 21.3] vs. **11.3** [8.8, 14.3] *p* = 0.713
**Minor errors, %**	**11.5 [8.3, 15.7] 27 studies, I^2^: 95%**	**10.6** [6.4, 17.3] 15 studies, I^2^: 94%	**12.2** [8.0, 18.3] 12 studies, I^2^: 95%	**10.0** [4.8, 19.6] 6 studies, I^2^: 98%	**8.5** [6.8, 10.7] 27 studies, I^2^: 84%	**12.6** [7.8, 19.6] vs. **10.6** [7.0, 15.7] *p* = 0.585
**Total errors , %**	**25.4 [19.5, 32.4] 28 studies, I^2^: 97%**	**24.8** [17.3, 34.3] 15 studies, I^2^: 94%	**26.1** [17.4, 37.2] 13 studies, I^2^: 98%	**21.8** [10.5, 40.0] 6 studies, I^2^: 98%	**21.5** [17.4, 26.2] 28 studies, I^2^: 94%	**29.9** [19.5, 42.8] vs. **21.5** [16.4, 27.7] *p* = 0.189

**Notes.**

a In quotation accuracy studies, indirect references are invariably counted as minor errors. Therefore, subtracting secondary references from the sum of errors (where possible) does not change the figures for major errors.

When we subtracted indirect references from minor and total quotation errors (as far as studies allowed for differentiating indirect references from other quotation problems) the figures fell slightly: minor quotation errors averaged 8.5% and total quotation errors amounted to 21.5% (Table 2).

In our main analysis, we left out each study at a time: the estimates for total quotation errors ranged between 23.4% (after studies 12 or 29 were dropped from the analysis) and 26.3% (when studies 16 or 17 were left out), indicating that no single study dominated the analysis.

In a subgroup analysis we found no difference between surgical and non-surgical specialties: the total quotation rate among nonsurgical specialties amounted to 25.4 [18.7, 33.5] while the summary estimate was 25.5 [14.3, 41.3] for the seven studies from surgery and orthopedics (*p* = 0.992).

**Figure 5:**
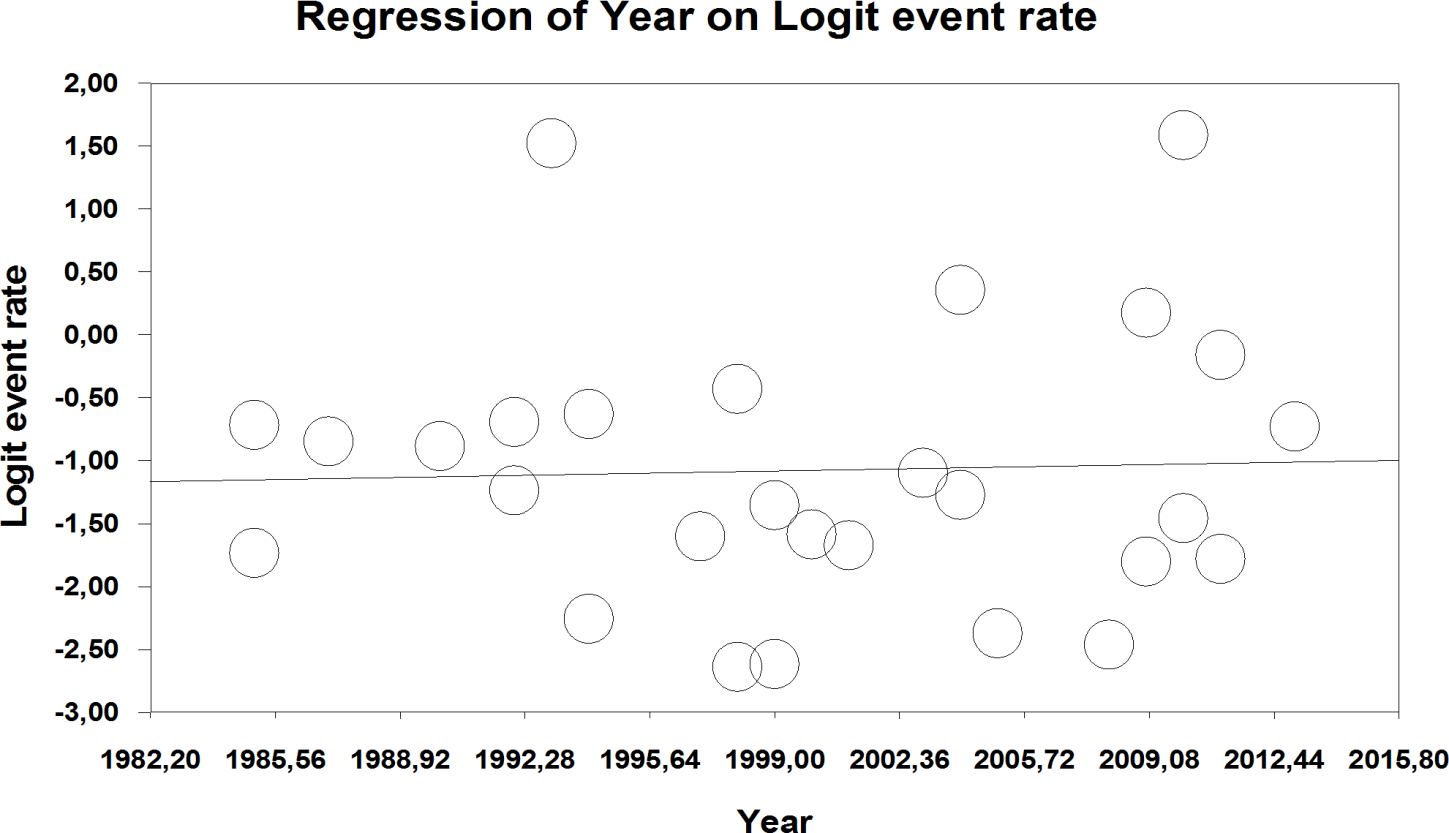
Meta regression: total quotation errors on publication date of study included in the present meta-analysis (main analysis).

No secular trend emerged from a meta regression of total quotation error rate on year of publication (mixed effects regression). In fact, as displayed in Fig. 5, there was a small positive slope of 0.0050 over time (−0.038, 0.0476; *p* = 0.817).

### Risk of bias analysis

Summary estimates for low risk of bias studies were larger than for high risk of bias studies (see Table 2), e.g., 29.9% [19.5–42.8] vs. 21.5% [16.4–27.7] with respect to total quotation errors (*p* = 0.189).

A funnel plot (Fig. S12), Eggers test (*p* = 0.242), and a trim and fill procedure revealed no sign of publication bias (main analysis, total quotation errors).

There was no indication that conflicts of interests may have introduced bias among the studies selected.

### Additional analysis

Three studies (Kolbitsch, Hörmann & Benzer, 1997; Porrino, Tan & Daluisiki, 2008; Rastegar & Wolfe, 2002) investigated whether a certain paper had been correctly referenced. Kolbitsch and co-workers (1997) reported on 5 major errors among 32 references to an anesthesiological paper from 1973 and found that only 13 references were in complete agreement, leaving approximately 60% of all references fraught with problems. Porrino, Tan & Daluisiki (2008) published a major error rate of one third (53/150) regarding an article about hand surgery and a minor error rate of 23/150. Finally, Rastegar and co-workers (2002) have found a total error rate of 14/121 references to one article from the field of primary care medicine.

## Discussion

This study yielded two main results: firstly, all studies included reported a substantial percentage of major and total quotation errors. Secondly, variability among the studies investigated was large.

Values for incorrect quotations varied, but we estimate that, on average, readers of scientific articles in medicine should be aware that there are problems with a sizable part—one fourth—of all references. According to our computation there is an error in the proper sense in every fifth quotation, roughly half of them so severe that they are not at all in accordance with what the authors claimed: in an average article with 50 references, based on our figures, about six are completely wrong.

Our estimate is similar to the median 20% quotation errors reported by Wager & Middleton (2008) in their review of studies until 2007.

### Heterogeneity and homogeneity of results

Variability among the studies analyzed was substantial, as indicated by large I^2^-values throughout our meta-analyses. While four sensitivity analyses, two subgroup analyses (including a risk of bias analysis) and one meta-regression indicate that our main findings are robust, they did not sufficiently elucidate the sources of heterogeneity in this sample of studies. The design of the studies, however, is straightforward, and with an average kappa of .76—if reported (Goldberg et al., 1993; Schulmeister, 1998; Reddy et al., 2008; Buijze et al., 2012; Luo et al., 2013)— the interrater reliability is sufficient according to the criteria of Landis & Koch (1977). In the only study differentiating kappa for major and minor errors, Reddy and co-authors (2008) showed that reliability was low for minor errors (*κ*0.26), indicating that subjective factors may account for some of the variability in our results regarding minor (and thus total) errors.

Different authors may have applied different approaches to identifying quotation errors—beyond the definition of major or minor errors. For example, in our own study (Mertens & Baethge, 2011) we considered a quotation correct even if it was not logically accurate, as in this sentence: “for a long time psychiatrists have debated about × (references).” In the strict sense, such a sentence demands a reference that supports the claim that psychiatrists, for a long time, debated on *x*. We deemed it correct, however, if the references were examples of such a discussion, thus supporting the meaning of the claim. From the studies included in this meta-analysis, as far as they presented examples of what they considered correct, we conclude that this was the prevailing approach to evaluating quotation accuracy.

In summary, we believe that the variability measured in our meta-analyses largely reflects true differences in quotation error rates among the samples under investigation—at least for major errors. It is, therefore, important to note that the majority of studies reported high rates of quotation errors suggesting that quotation errors are a considerable phenomenon in medical journal articles, even if numbers differ. For example, only six out of 28 studies reported total error rates below 10% (Hansen & McIntire, 1994; Schulmeister, 1998; Lawson & Fosker, 1999; Pieters, Ceysens & De Heyn, 2001; Gupta et al., 2005; Reddy et al., 2008), and none below 6.7%. The latter figure translates into one misleading reference out of every fifteen citations. Thus, the heterogeneity found in this sample is entirely among investigations indicating significant rates of quotation errors. While the evidence for a substantial number of quotation errors in medical journal articles is high, heterogeneity is high and the estimates from our meta-analyses have to be viewed with extreme caution and to be taken as what they are: weighted averages from a heterogeneous field, calculated in order to describe the average magnitude of the effect, not to put a final number to a phenomenon.

### Conservative estimate

It seems plausible that our findings represent a cautious estimate of quotation problems: the analyses summarized here are restricted to the question whether a source cited by the authors supported their statement, but it is possible that a source is, technically, supportive but inadequate nevertheless. For example, the content of a cited paper turns out to be irreplicable, or does not stand up to further scientific scrutiny in other studies. Or the paper contained plain errors: Hauptmann and co-authors (2014) found that 4.2% of original and review articles in high impact factor journals were followed by an error report, with major errors in about one fourth of such articles. Other researchers have made the sobering discovery that even an obviously absurd study from the BMJ Christmas issue has been referred to, in one third of its citations, as if it was serious science (Felding, Jørgensen & Hróbjartsson, 2009).

Another problem is even more difficult to investigate: the lack of a quotation where one is needed: e.g., plagiarizing or not mentioning important earlier work.

The studies selected differed in their classification of indirect references. As a result, indirect references were not counted as errors in some studies. Whether indirect references are errors, however, depends on the accuracy of the review cited. We consider indirect references—and also citations of statements from discussion sections —problematic for three reasons: (1) Because they make it more difficult for the reader to follow a particular statement. (2) Errors may easily be propagated (“Chinese whispers”). (3) Authors may be acknowledged for ideas they did not develop themselves. Often times, on the other hand, such quotations are not erroneous. In fact, with the rise of systematic literature searches and more sophisticated methods of summarizing research results reviews can be helpful for authors in their evaluation of content they are about to refer to. Further, if many original articles can be cited readers may get a better picture from referencing a review. Nevertheless, authors should not cite systematic reviews at the expense of crediting researchers for important papers.

### What are the consequences of quotation errors?

One consequence of high error rates is obvious: readers can not be sure that a quotation adds weight to a scientific statement. Although unsupported claims may create a false sense of certainty in readers and may seriously mislead scientists, many experienced researchers are aware of a high degree of uncertainty in scientific articles. However, doctors more occupied with patient care than with critically assessing scientific articles are probably less familiar with the uncertainties in medical research. They, and also the general public, have to rely on sound scientific writing.

While the findings do not cast a positive light on scholarly writing in medicine and quotation errors may undermine the trust of the medical as well as the general public in medical science, the damage to the scientific process is hard to judge. For example, it is important to what extent a misleading quotation is essential for the thrust of the paper or whether such a mis-quotation is merely a reverence to scientific tradition, a matter of duty, in an otherwise sound study or review. A paper is not automatically invalidated because it contains quotation errors. In fact, it is conceivable that quotation errors are not particularly momentous: the progress of science may be dependent on completely different factors, such as ingenuity, associative rather than ordered thinking, or stamina in pursuing off-mainstream ideas, whereas building a water-proof argument in the light of former science by meticulously citing earlier publications may be not instrumental to progress. The opposing view has been expressed by Gupta et al. (2005): “References are akin to mortar, which not only binds the bricks together in a wall but also lends it the most vital things, i.e., strength and durability.”

Future research should investigate the extent to which false quotations are important for the claim of the ctiting paper. In the same vein, it may be instructive to compare the rate of quotation errors in breakthrough papers with more modest publications. Whilst we are not aware of such data for medicine, in the field of economics, Harzing (2002) has shown that wrong quotations can play an important role in creating a scientific “myth.” Table S1 lists possible research topics in this field and Table S2 summarizes methodological suggestions for future research.

It is unclear how much quotation errors distort citation-based evaluation metrics in medicine. One reason is that it is unknown whether quotation errors occur at random or whether they are associated with defined characteristics of, for example, articles, authors, topics, or more technical aspects, such as number of references. The studies included in this overview did not unanimously report certain predictors. It seems plausible, however, that the effect of quotation errors is becoming smaller the larger the number of references in an evaluation. For example, false quotations may cancel each other out in a comparison of universities.

### What may be the reasons for quotation errors?

Several authors of studies on quotation errors discuss doubts that writers wrongly refering to a paper have actually read it (e.g., Eichorn & Yankauer, 1987; Gosling, Cameron & Gibbons, 2004; Awrey et al., 2010). For the field of physics, Simkin & Roychowdhury (2005), using propagation of misprints in bibliographical references as a model, estimated that about 70–90% of citations are copied from other papers’ reference lists and that the original papers have not been read. Whereas propagation of incorrect bibliographic information does not necessarily mean that a paper has not been read (even though it is likely cited from the wrong source) it is plausible that such an effect, however large, exists in medicine too.

In part from our own reading experiences we believe authors can interpret papers they read as they would like to rather than what they really say (Wynder, Higgins & Harris, 1990; Epley & Whitchurch, 2008). This (often unconscious) bias may also contribute to erroneous quotations, particularly when time constraints and pressure to publish play a role too. Also, wrong quotations may easily creep in if authors want to credit a colleague or if they feel the need to fit in a reference that seems politically useful, adds spin or was asked for by a reviewer or journal editor.

Finally, original sources may be reported in such a distorted way (Boutron et al., 2010) that superficial readers mistake the meaning of a paper and quote it inaccurately.

Another contributing factor may be citing style: in medicine, similar to the natural sciences, references do not come as explanatory footnotes or endnotes but are restricted to the reference list at the end of the text. In contrast, when authors from the humanities and social sciences refer to a source they often use footnotes or endnotes to elaborate on the reference while the reference list itself merely serves bibliographical needs. In theory, such a citing style makes it much harder to get away with misleading quotations. We are not aware, however, of a study testing the hypothesis that quotation errors are more frequent in medicine than in the humanities.

### How do these findings compare with other academic fields?

Studies in other fields report numbers similar to those in this meta-analysis. In ecology, several studies have been conducted: Todd et al. (2007), in an investigation of 306 references, found that 7.2% did not support the original statement (a major error in the definition of this article), 11.1% were “ambiguous” (minor errors), and 5.6% were “empty” references (secondary or indirect references in our terminology). Subsequently, Drake and co-authors (2012) and Teixeira and co-authors (2013) reported even higher figures. A similar picture emerges from studies in the fields of marine biology (Todd et al., 2010), geography (Haussmann et al., 2013), and veterinary medicine (Hinchcliff et al., 1993).

In a meticulous case study of referencing problems in a group of 60 papers from the economics literature (Harzing, 2002), Harzing has documented many examples of misquotations. This paper is exceptional in that it presents a qualitative approach.

### What can be done?

The most important consequence does not concern authors but readers of medical journal articles: similar to other scientific aspects of medical articles, for instance, the accuracy of design and statistical procedures or of numerical presentations, accuracy of quotations can not be taken for granted and scepticism is in order.

Apart from such universal conclusions and from the general advice to authors to quote more accurately, many authors have brought forward suggestions for improvements. These suggestions can be divided into proposals for authors, who carry, it is widely agreed, the final responsibility for quotation accuracy (e.g., Eichorn & Yankauer, 1987; Buchan, Norris & Kuper, 2005; Reddy et al., 2008) and proposals for journals. The following list contains examples of others’ and our own proposals. The proposals are diverse and, therefore, not necessarily inherently consistent. It is also important to note that, to our knowledge, none of the following measures can claim a high level of evidence, such as RCTs.

**For authors:**

• In preparation of the manuscript: Authors should check all references (as proposed, for example, in Lowry, 1985; Porrino, Tan & Daluisiki, 2008; Buijze et al., 2012). Statements should be verified against original papers, not indirect sources. Citation of original material is preferred over abstracts and narrative reviews (e.g., Lowry, 1985; Gupta et al., 2005; Buijze et al., 2012; International Committee of Medical Journal Editors, 2014).
• In writing the manuscript: groups of references at the end of a sentence or paragraph should be avoided. Instead, references should be placed close to the formulation or word they refer to e.g., Eichorn & Yankauer (1987). The best available support should be cited, for instance, a Cochrane review and not a single trial, or an RCT and not observational studies (Buijze et al., 2012).
• On submission of the manuscript: when submitting a manuscript authors should declare they have checked all references for accuray and have used primary references instead of indirect ones (e.g., Goldberg et al., 1993). Authors should submit copies of all articles they cite (e.g., Fenton et al., 2000).

**For journals:**

• In the requirements for referencing: Restriction of the number of references so that it is easier for authors to maintain an overview over what they cite (e.g., Lee & Lee, 1999; Fenton et al., 2000; Buijze et al., 2012). Editors may consider whether certain statements really need one or even more than one reference, particularly in discussion sections. Use of footnotes or endnotes instead of merely reference lists. Using Harvard citation style (author, date) instead of Vancouver style (numbered) may make it easier for peers to detect errors.
• In evaluating manuscripts: Editors (e.g., Lee & Lee, 1999; Singh & Chaudhary, 2009) and Reviewers (e.g., Schulmeister, 1998; Lukić et al., 2004) should check selected references.
• In communicating with authors: Editors should inform authors that accuracy in quotations is expected (Goldberg et al., 1993) and checked; they could contact authors and ask for confirmation that they have read cited papers (Wright & Armstrong, 2008). It may be counterproductive to ask authors to cite only relatively recent papers because that may be one reason for secondary references (Schulmeister, 1998). Journals could institute a misquotations column presenting cases of quotation errors (De Lacey, Record & Wade, 1985; Fenton et al., 2000).

Whereas some of the proposals seem unrealistic, at least in the short run (e.g., restriction of the number of references, introduction of footnotes in medical articles, submitting cited articles), or may merely antagnoize authors (misquotation column), others may be immediately instituted: random checks by editorial staff and reviewers or placing references next to the claim, for example.

### Limitations of the present study

We may have missed studies in our literature search. While we searched Medline, PubMed Central, and Web of Science (in a reference search) and hand-searched all selected articles for further studies and while we found all studies Wager & Middleton (2008) included in their overview (using Medline *and* Embase), using more databases may have yielded additional studies. It seems improbable, however, that further studies may have meaningfully changed the main finding: under the unlikely assumptions of missing 39 studies (more than the sum of studies found in our literature search), each of which reporting a total quotation error rate of only 5% (lower than the lowest estimate reported in the studies selected for this review), the summary estimate would still amount to a substantial quotation error rate of 10%. Also, there was no indication for publication bias, which seems plausible because there is no negative result in the strict sense in this area of research.

The studies cover a broad range of medical specialties, but they are not representative for medicine in general. There are, for instance, no studies from urology, from forensic medicine, or from physiology. Finally, except for two Dutch and one German paper, all studies dealt with English literature which is why we can not claim representativeness for other languages.

## Conclusion

In reviewing more than two dozen studies we found that quotation errors are common in medical journal articles. For the first time, this study presents an estimate of quotation inaccuracy. Even though there was considerable variation across studies the finding of substantial inaccuracy proved robust and surprisingly similar in several sensitivity and subgroup analyses. While findings of studies selected were heterogenous, the main finding is homogeneous.

Although quotation errors undermine trust in journal articles it is difficult to determine how dangerous they are for clinical medicine and for the progress of science. Future research should focus on the significance of quotation errors for the creation of medical myths and for scientific progress. For the time being readers should be sceptic of quotations in medical articles, and authors as well as editors and reviewers can improve the situation by meticulously checking what is cited.

## Supplemental Information

10.7717/peerj.1364/supp-1 Supplemental Information 1 PRISMA checklist

10.7717/peerj.1364/supp-2 Table S1 Topics in quotation accuracy research

10.7717/peerj.1364/supp-3 Table S2 Methodological suggestions for fututre research

10.7717/peerj.1364/supp-4 Figure S1 Total quotation errors, based on references, restricted to 1 error per reference

10.7717/peerj.1364/supp-5 Figure S2 Total quotation errors, based on references, unrestricted

10.7717/peerj.1364/supp-6 Figure S3 Total quotation errrors, quotation based, unrestricted

10.7717/peerj.1364/supp-7 Figure S4 Data on major errors, based on references, restricted to 1 error per reference

10.7717/peerj.1364/supp-8 Figure S5 Major quotation errors, based on references, unrestrited to 1 error per reference

10.7717/peerj.1364/supp-9 Figure S6 Major quotation errors, based on quotations, unrestricted to 1 error per quotation

10.7717/peerj.1364/supp-10 Figure S7 Minor quotation errors, based on references, restricted to 1 error per reference

10.7717/peerj.1364/supp-11 Figure S8 Minor quotation errors, based on references, unrestricted to 1 error per reference

10.7717/peerj.1364/supp-12 Figure S9 Minor quotation errors, data based on quotations, unrestricted to 1 error per quotation

10.7717/peerj.1364/supp-13 Figure S10 Total quoatation errors (main analysis) without indierect references

10.7717/peerj.1364/supp-14 Figure S11 Minor quotation errors (main analysis) without indirect references

10.7717/peerj.1364/supp-15 Figure S12 Funnel plot (total quotation errors, main analysis)

## Additional Information and Declarations

### Data Availability

The following information was supplied regarding data availability: Raw data are present in the paper and its supplemental materials. The methods, tables, and figures contain all data necessary to reproduce all results in this paper.
