# Supplementary material for: Quotation accuracy in medical journal articles—a systematic review and meta-analysis
Source: PeerJ. 2015 Oct 27;3:e1364. doi: 10.7717/peerj.1364 (PMC4627914; doi:10.7717/peerj.1364)
Supplement: Table S1 [file peerj-03-1364-s002.docx]

**Supplementary table 1. Topics in quotation accuracy research**

| Quotation accuracy in medical literature beyond English, Dutch, and German |
| --- |
| Are certain statements particularly prone to quotation errors (i.e., prevalence statements, group references)? |
| Quotation accuracy in medical specialties not covered so far, e.g., urology, forensic medicine, physiology |
| Does quotation inaccuracy undermine research or is it unimportant for the progress of science?  Approaches:   - Comparison of research accuracy between important medical publications (for example, citation classics or classics in the opinion or experienced peers) and average papers - Qualitative investigation of the importance of quotation errors for the thrust of an article - Are scientific myths created by quotation errors (similar to the investigation of Harzing (38))? |
| Are different kinds of quotations, e.g. footnotes, associated with different rates of quotation errors? |
| What is the quotation error rate in humanities and social science? |
