# Supplementary material for: Quotation accuracy in medical journal articles—a systematic review and meta-analysis
Source: PeerJ. 2015 Oct 27;3:e1364. doi: 10.7717/peerj.1364 (PMC4627914; doi:10.7717/peerj.1364)
Supplement: Table S2 [file peerj-03-1364-s003.docx]

**Supplementary table 2. Methodological suggestions for future research**

| In addition to major, minor and total quotation error rates results for different groups of quotation errors, e.g., indirect references should be documented. |
| --- |
| Consider counting reference errors as quotation errors if the source can not be traced and, thus, the claim can not be checked. |
| Use random selection of references and quotations. |
| Use at least two independent raters. Document interrater reliability and the number of cases difficult to judge. |
| Present reference based and quotation based analysis. |
| Count all quotation errors and do not restrict quotation errors to one per reference/quotation. |
| Specifiy the approach to evaluating quotation accuracy: Liberal versus strict. A strict approach would mean that in the sentence „For a long time psychiatrists have debated about xy (references)“ quotations would be considered wrong if references were not about the discussion but only examples of papers that have played a role in the discussion. (Our advice is to evaluate quotation errors using a liberal approach and to evaluate the sense and not the logical structure of an approach.) |
