# Supplementary figures and images for: Quotation accuracy in medical journal articles—a systematic review and meta-analysis
Source: PeerJ. 2015 Oct 27;3:e1364. doi: 10.7717/peerj.1364 (PMC4627914; doi:10.7717/peerj.1364)

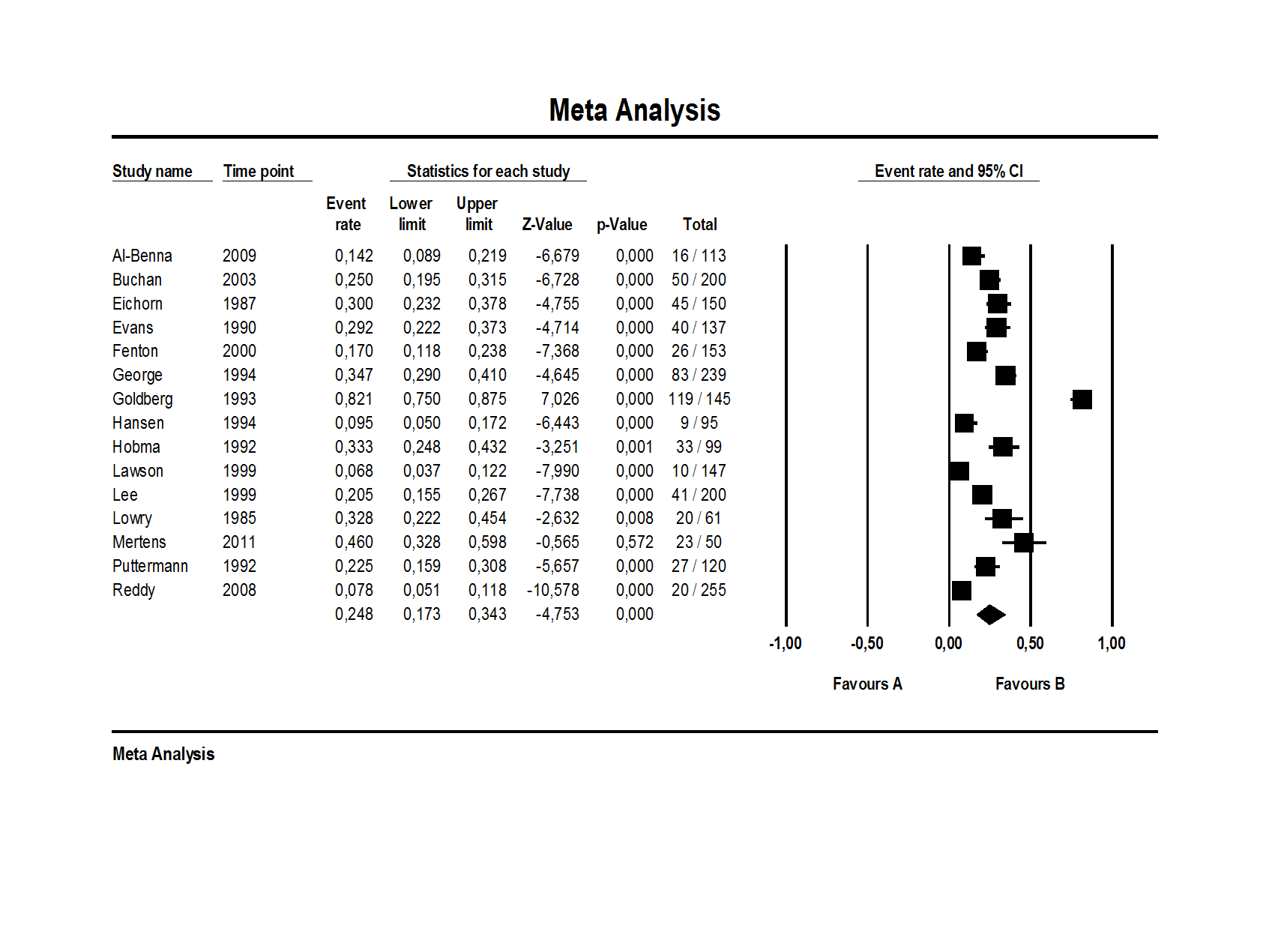

Supplement: Figure S1 [file peerj-03-1364-s004.png]

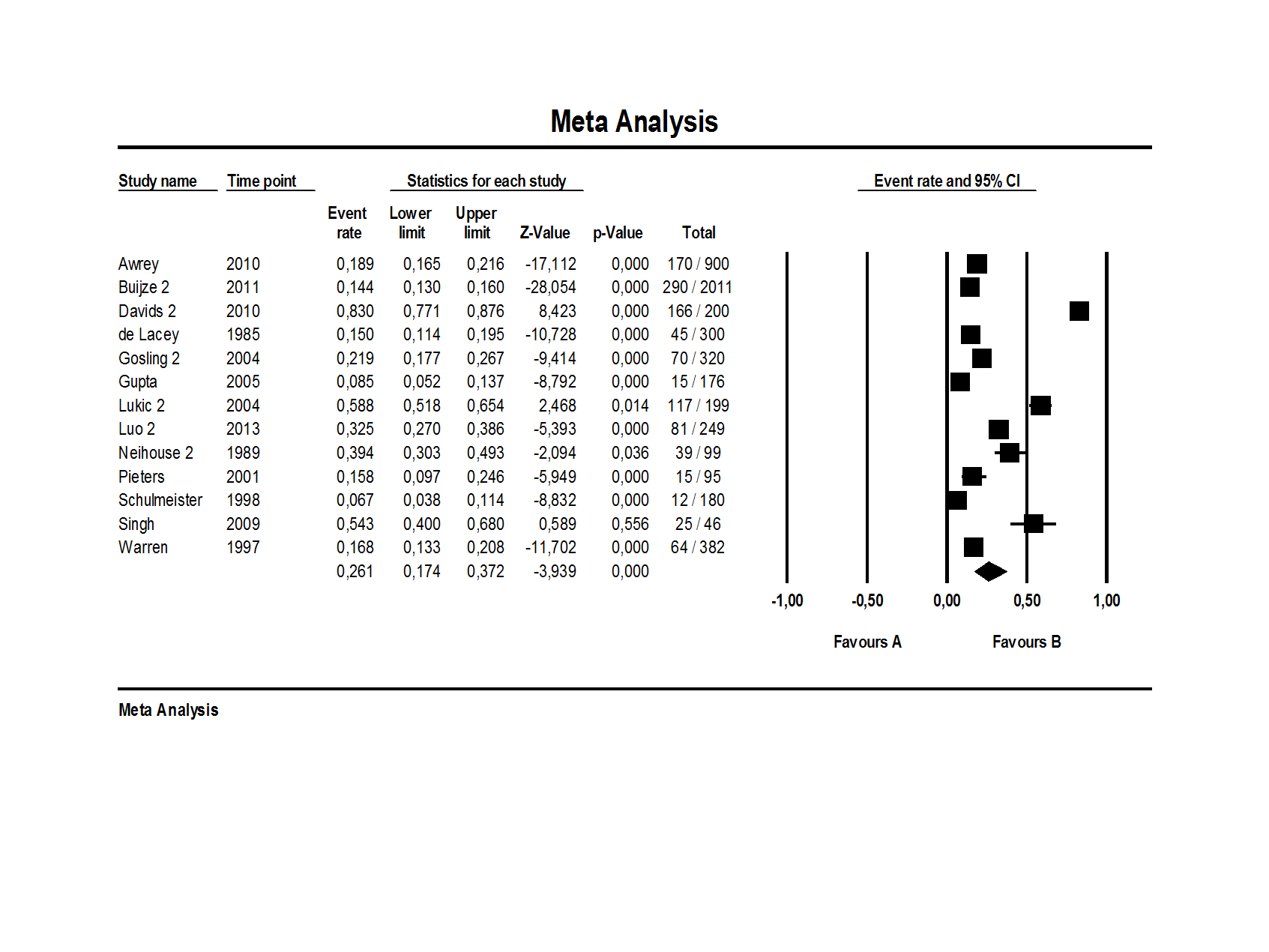

Supplement: Figure S2 [file peerj-03-1364-s005.png]

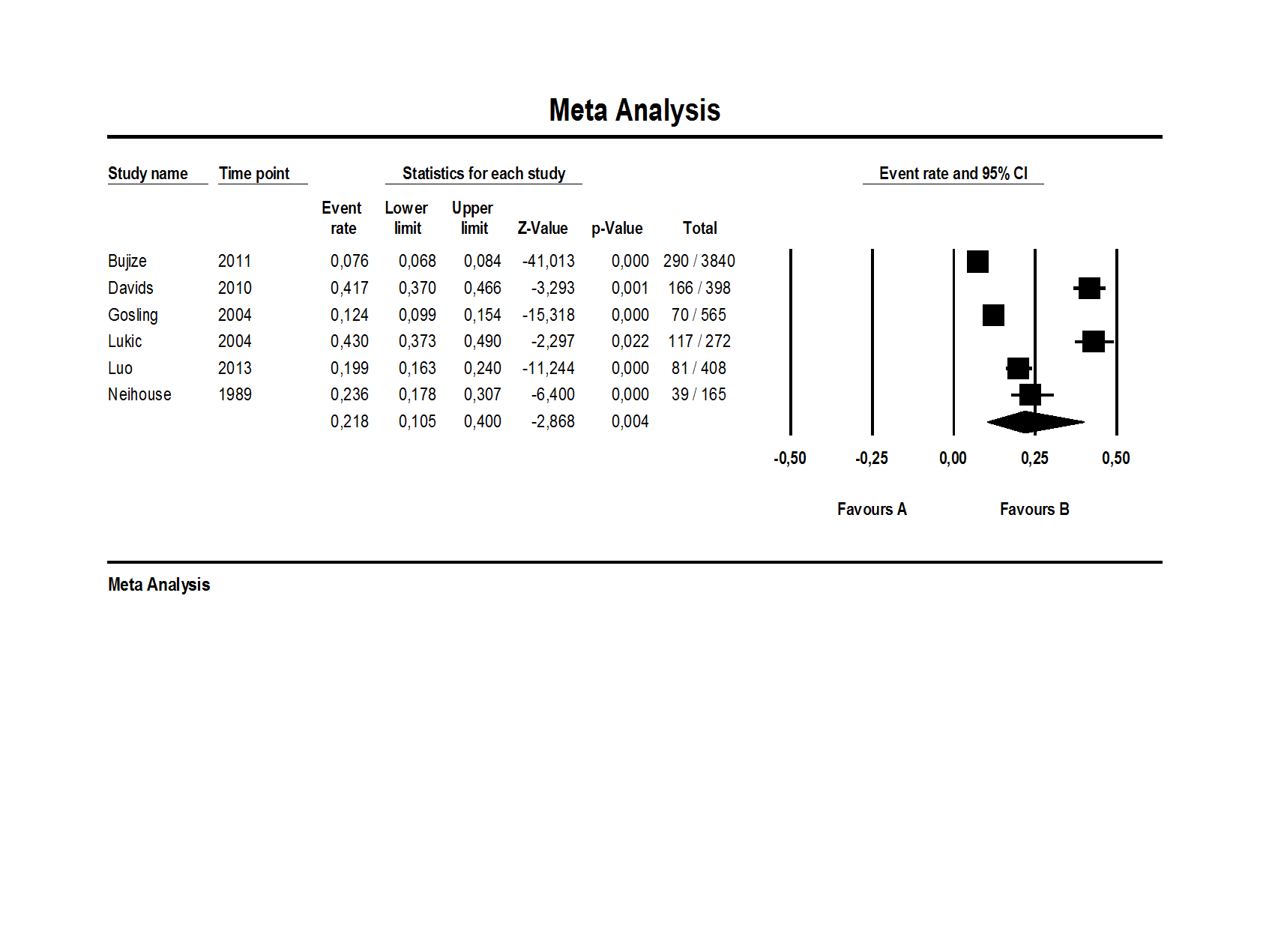

Supplement: Figure S3 [file peerj-03-1364-s006.png]

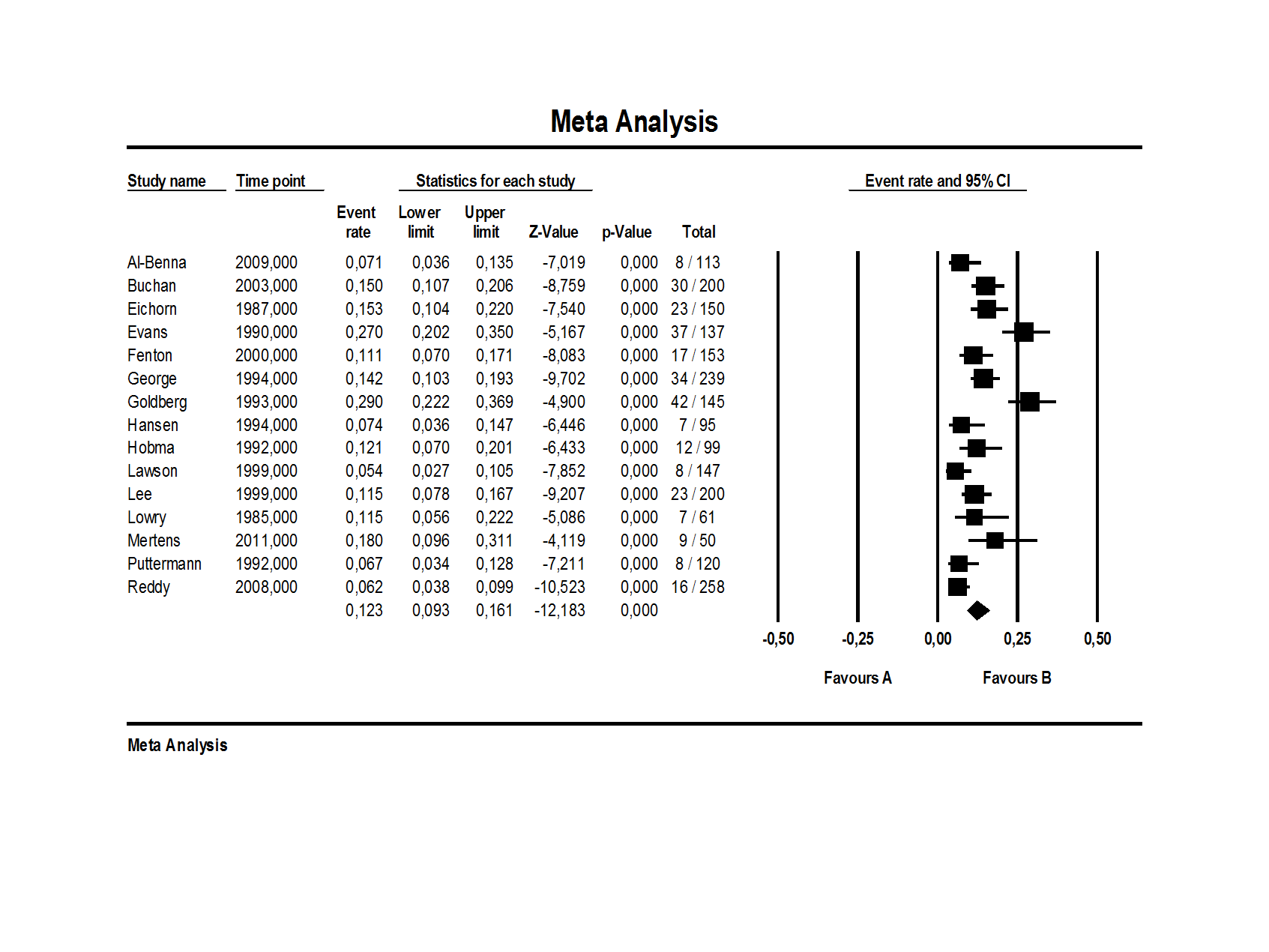

Supplement: Figure S4 [file peerj-03-1364-s007.png]

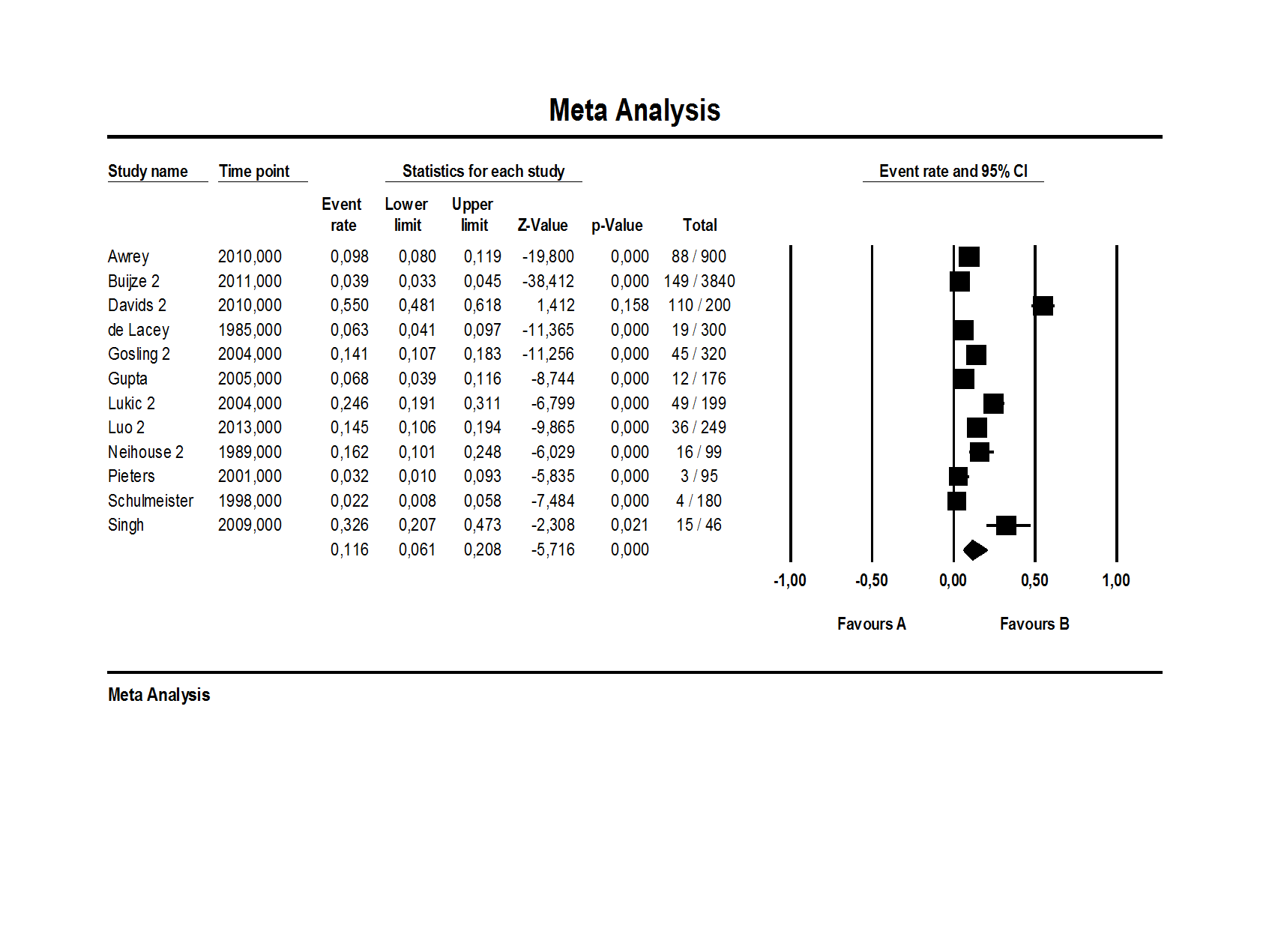

Supplement: Figure S5 [file peerj-03-1364-s008.png]

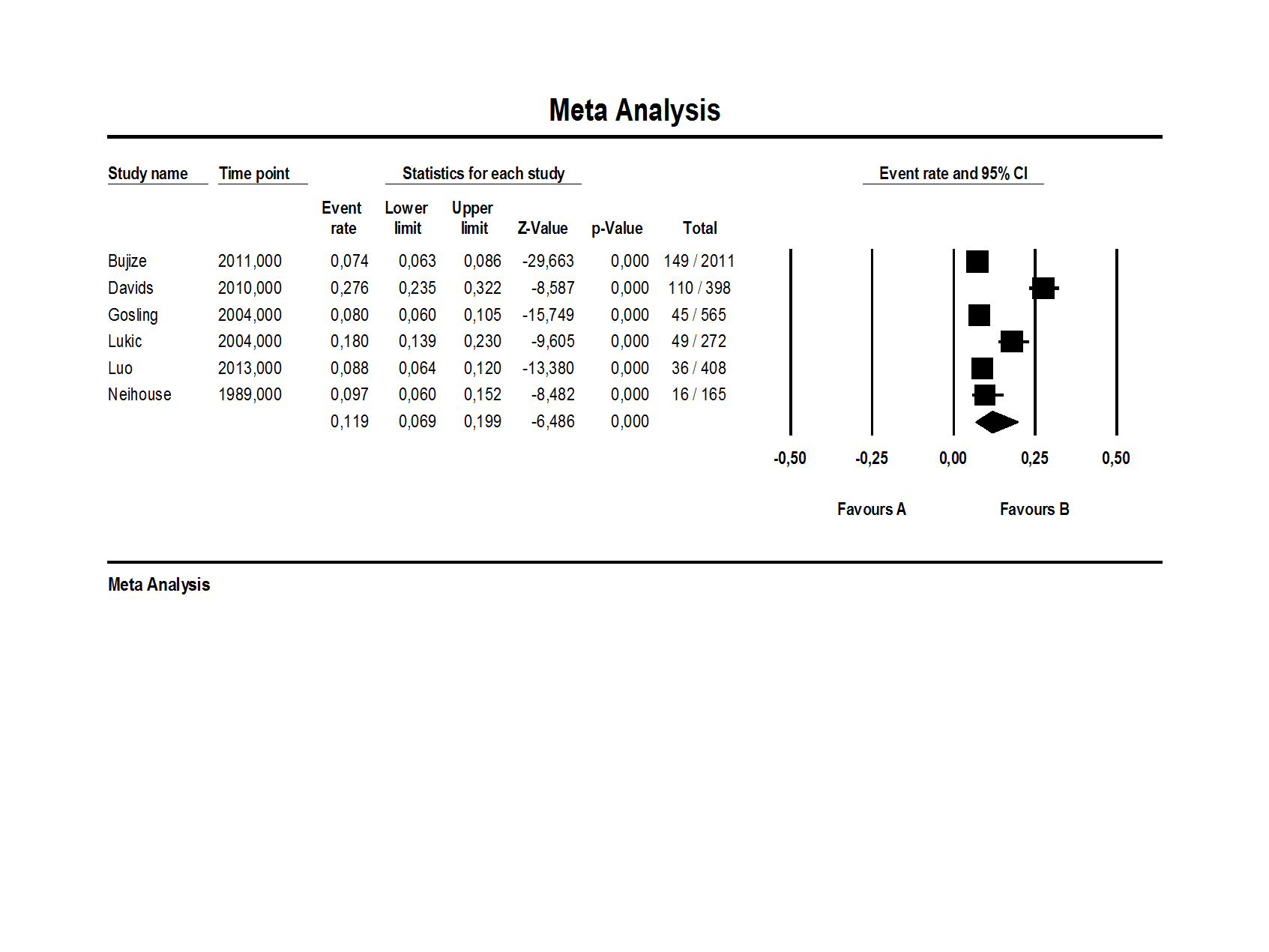

Supplement: Figure S6 [file peerj-03-1364-s009.png]

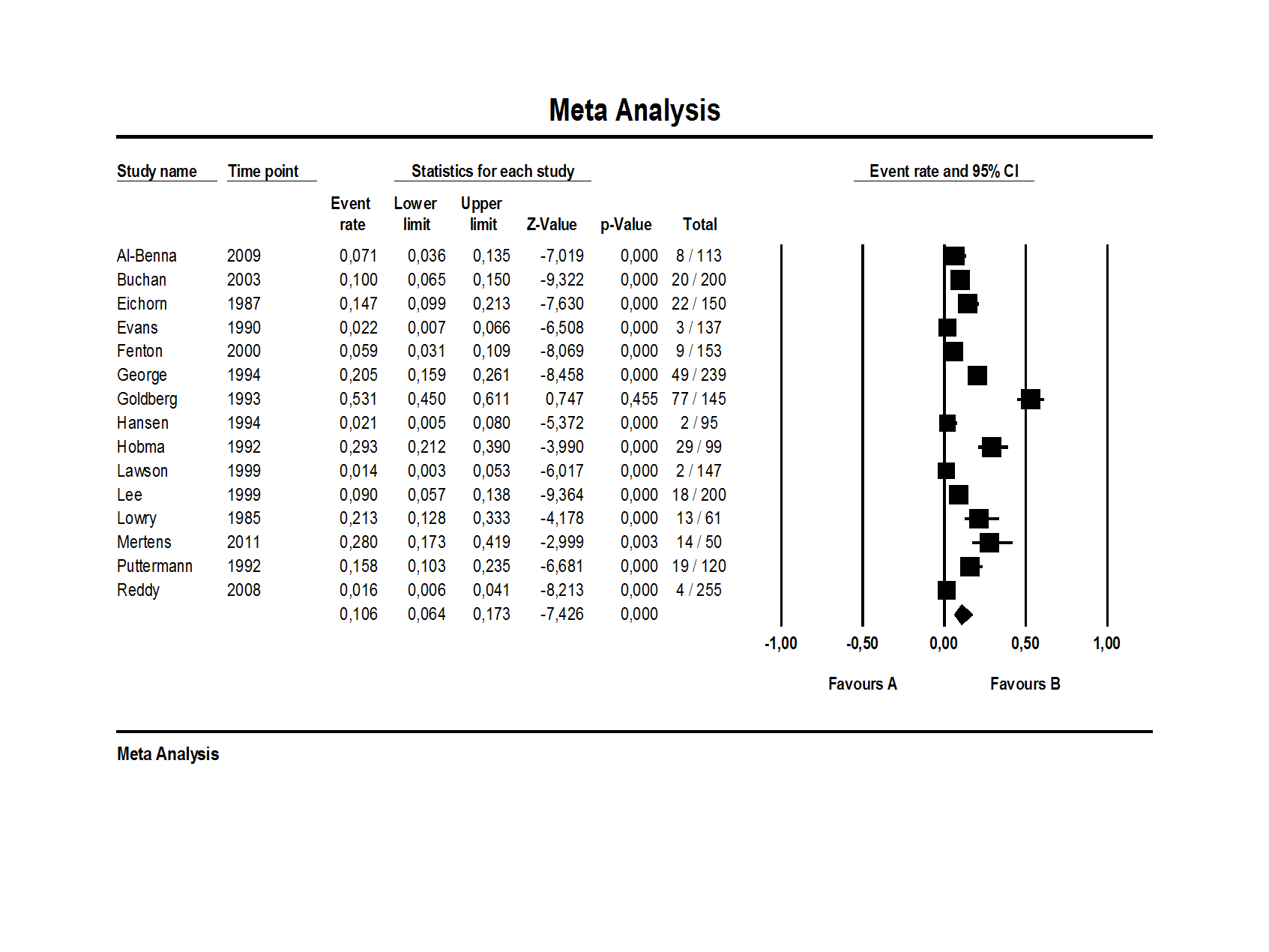

Supplement: Figure S7 [file peerj-03-1364-s010.png]

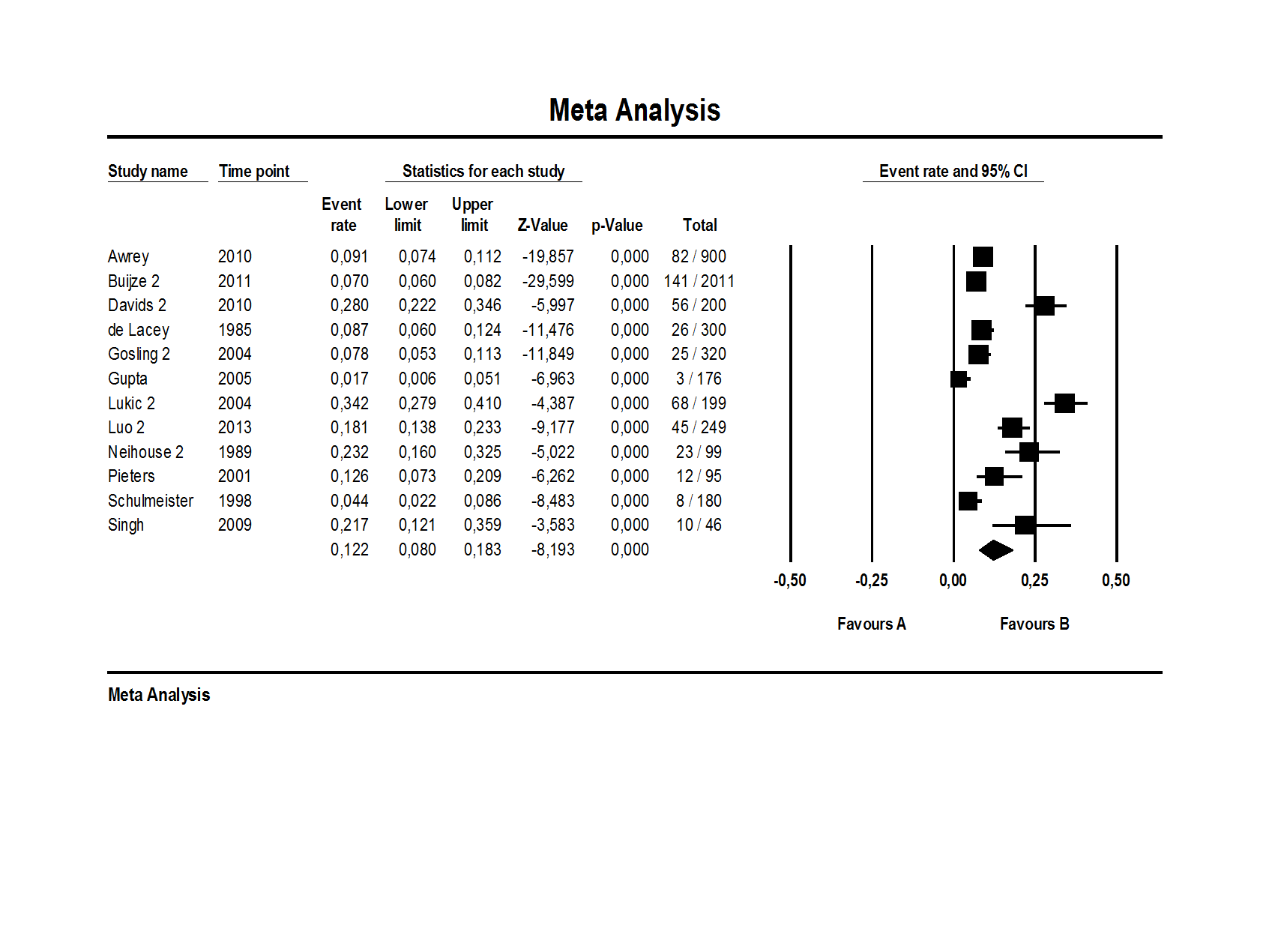

Supplement: Figure S8 [file peerj-03-1364-s011.png]

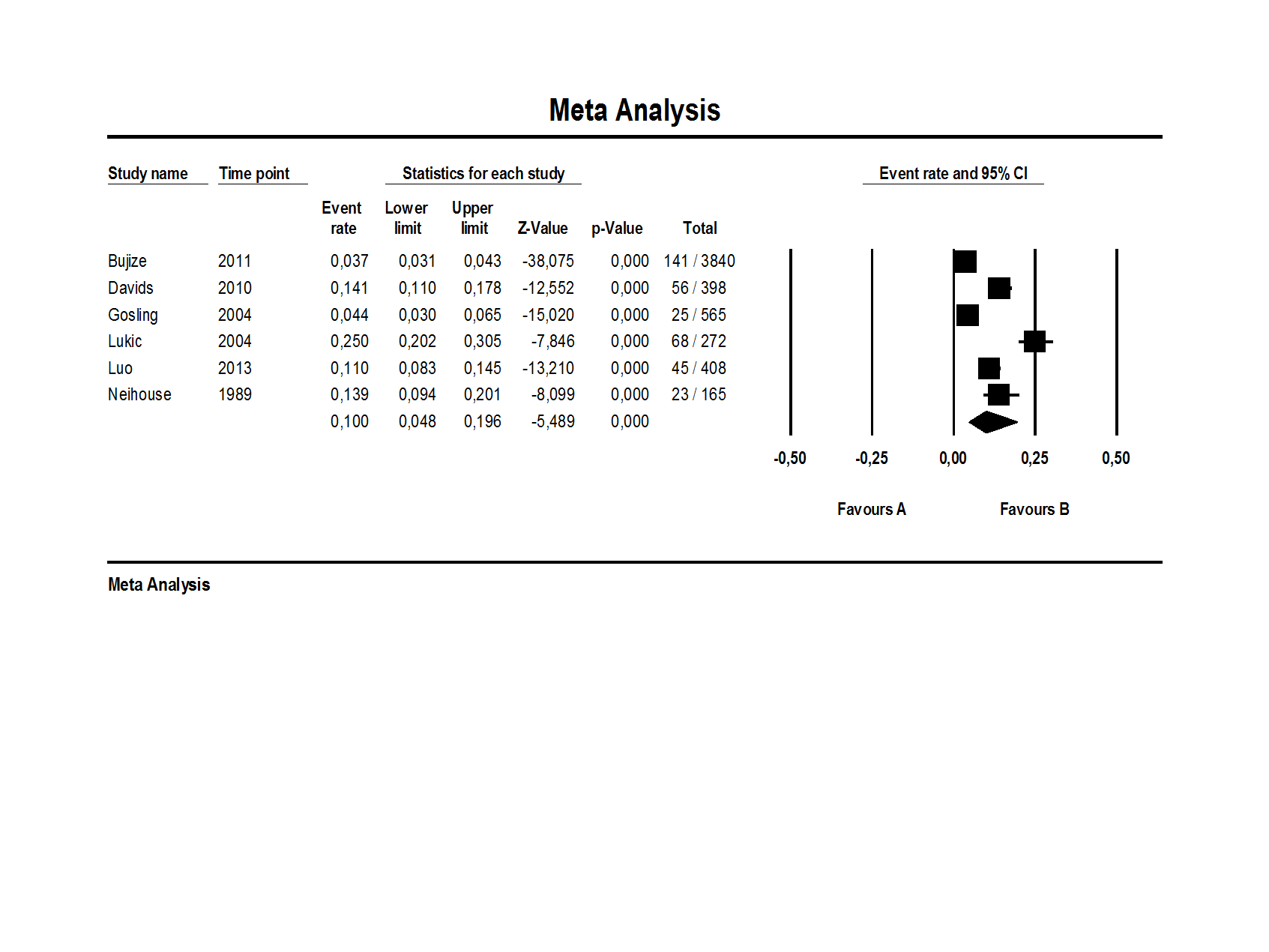

Supplement: Figure S9 [file peerj-03-1364-s012.png]

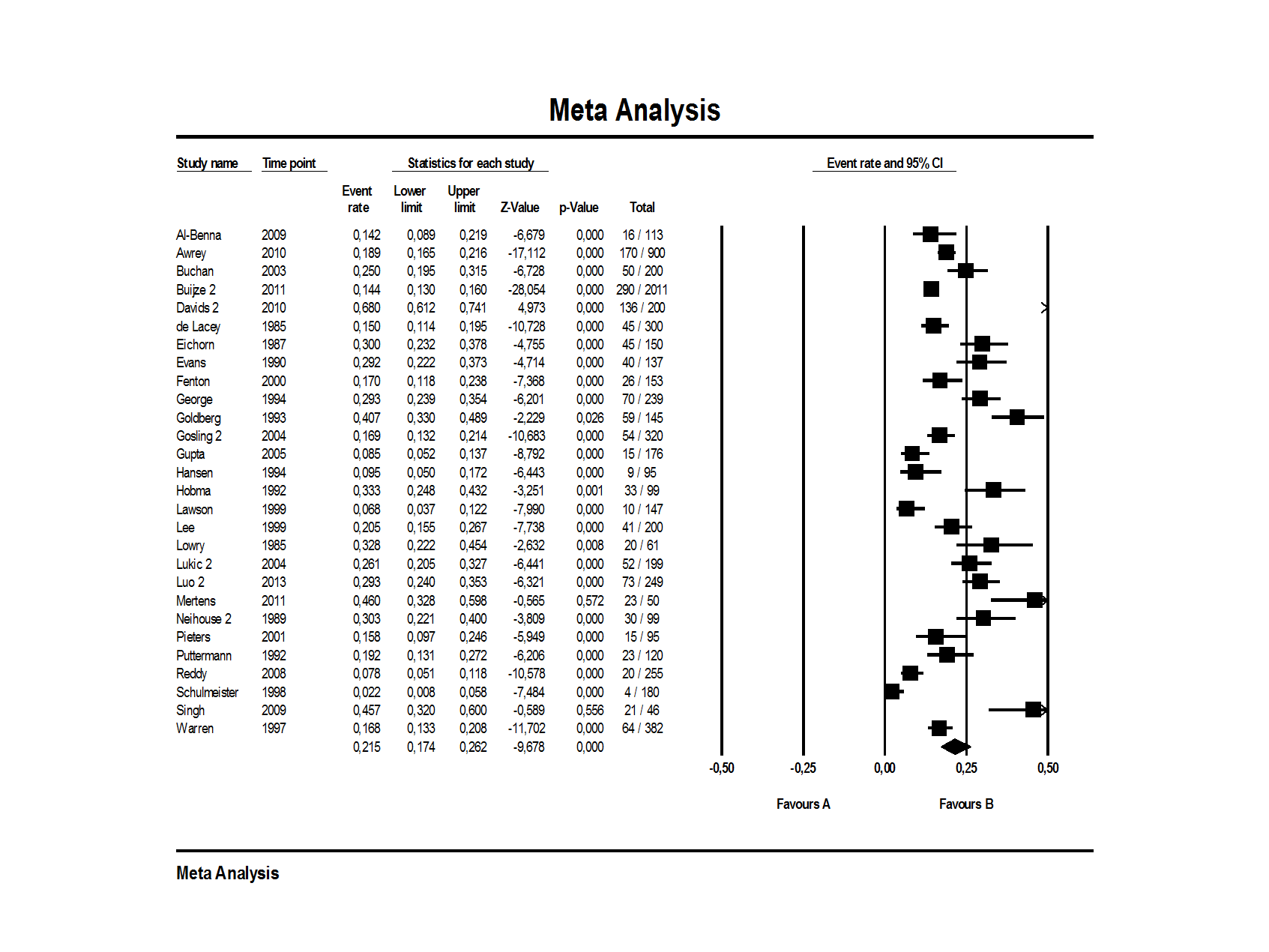

Supplement: Figure S10 [file peerj-03-1364-s013.png]

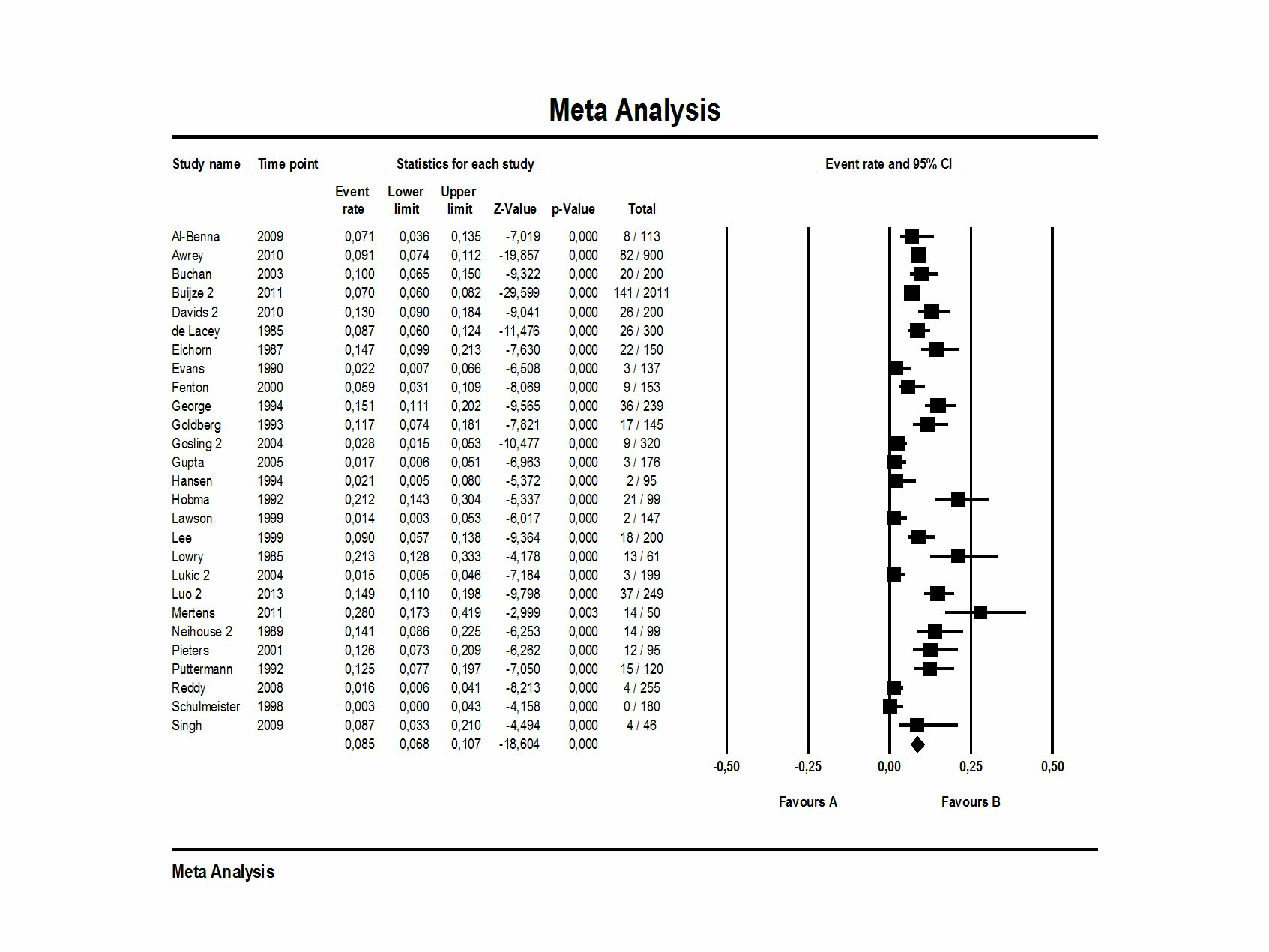

Supplement: Figure S11 [file peerj-03-1364-s014.png]

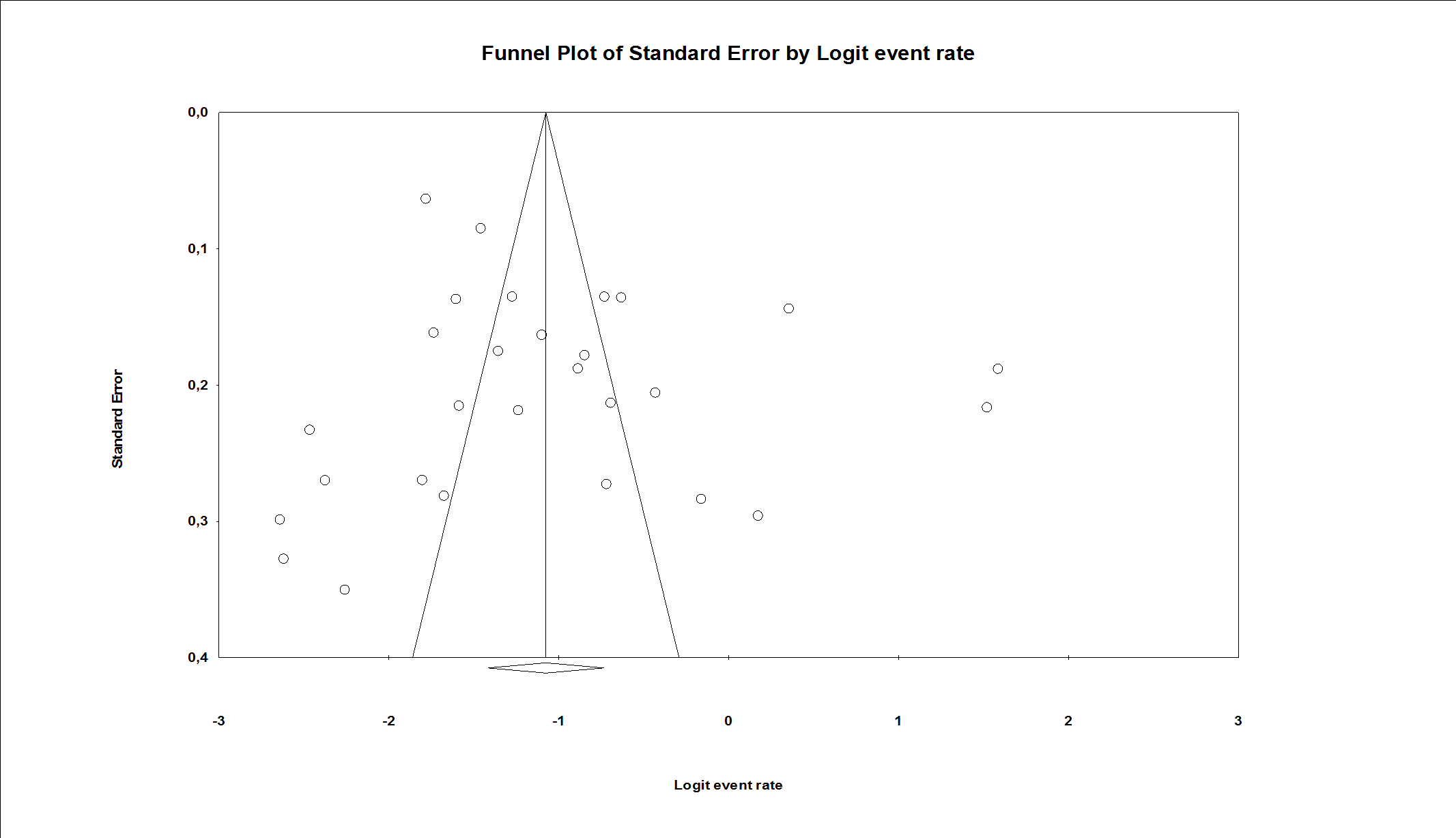

Supplement: Figure S12 [file peerj-03-1364-s015.png]
